# Enhancing prediction of supraspinatus/infraspinatus tendon complex injuries through integration of deep visual features and clinical information: a multicenter two-round assessment study

**DOI:** 10.1186/s13244-023-01551-1

**Published:** 2023-11-23

**Authors:** Yamuhanmode Alike, Cheng Li, Jingyi Hou, Yi Long, Jinming Zhang, Chuanhai Zhou, Zongda Zhang, Qi Zhu, Tao Li, Shinan Cao, Yuanhao Zhang, Dan Wang, Shuangqin Cheng, Rui Yang

**Affiliations:** 1 Department of Orthopaedic Surgery, Sun Yat-Sen Memorial Hospital, Sun Yat-Sen University, 107# Yan Jiang Road West, Guangzhou, 510120 Guangdong Province People’s Republic of China; 2 Department of Orthopaedic Surgery, Shenshan Medical Center, Sun Yat-Sen Memorial Hospital of Sun Yat-Sen University, Shanwei, People’s Republic of China; 3 The School of Biomedical Science, The Chinese University of Hong Kong, Hong Kong, People’s Republic of China; 4 The College of Information Science and Technology, Jinan University, Guangzhou, People’s Republic of China

**Keywords:** Deep learning, Machine learning, Rotator cuff injury, Two-round assessment

## Abstract

**Objective:**

Develop and evaluate an ensemble clinical machine learning–deep learning (CML-DL) model integrating deep visual features and clinical data to improve the prediction of supraspinatus/infraspinatus tendon complex (SITC) injuries.

**Methods:**

Patients with suspected SITC injuries were retrospectively recruited from two hospitals, with clinical data and shoulder x-ray radiographs collected. An ensemble CML-DL model was developed for diagnosing normal or insignificant rotator cuff abnormality (NIRCA) and significant rotator cuff tear (SRCT). All patients suspected with SRCT were confirmed by arthroscopy examination. The model’s performance was evaluated using sensitivity, specificity, accuracy, and area under the curve (AUC) metrics, and a two-round assessment was conducted to authenticate its clinical applicability.

**Results:**

A total of 974 patients were divided into three cohorts: the training cohort (*n* = 828), the internal validation cohort (*n* = 89), and the external validation cohort (*n* = 57). The CML-DL model, which integrates clinical and deep visual features, demonstrated superior performance compared to individual models of either type. The model’s sensitivity, specificity, accuracy, and area under curve (95% confidence interval) were 0.880, 0.812, 0.836, and 0.902 (0.858–0.947), respectively. The CML-DL model exhibited higher sensitivity and specificity compared to or on par with the physicians in all validation cohorts. Furthermore, the assistance of the ensemble CML-DL model resulted in a significant improvement in sensitivity for junior physicians in all validation cohorts, without any reduction in specificity.

**Conclusions:**

The ensembled CML-DL model provides a solution to help physicians improve the diagnosis performance of SITC injury, especially for junior physicians with limited expertise.

**Critical relevance statement:**

The ensembled clinical machine learning–deep learning (CML-DL) model integrating deep visual features and clinical data provides a superior performance in the diagnosis of supraspinatus/infraspinatus tendon complex (SITC) injuries, particularly for junior physicians with limited expertise.

**Key points:**

1. Integrating clinical and deep visual features improves diagnosing SITC injuries.

2. Ensemble CML-DL model validated for clinical use in two-round assessment.

3. Ensemble model boosts sensitivity in SITC injury diagnosis for junior physicians.

**Graphical Abstract:**

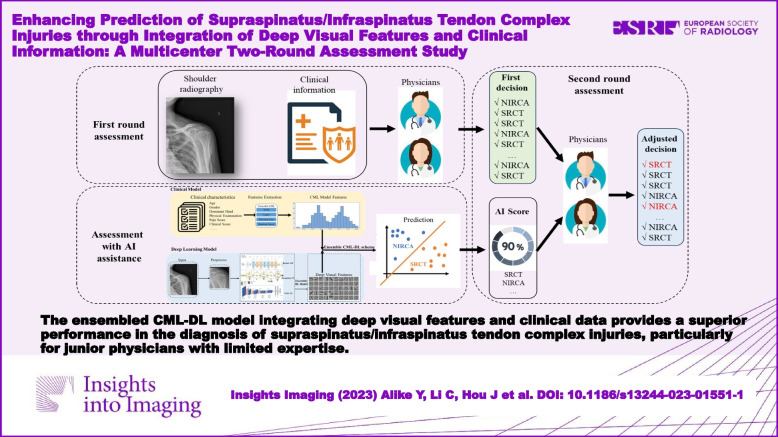

## Introduction

Rotator cuff tears (RCTs) are a prevalent and debilitating condition that affect millions of people worldwide [1]. RCTs are a common cause of shoulder pain and dysfunction and are often associated with significant reductions in quality of life and functional disability. The prevalence of RCTs increases with age, with some studies suggesting that up to 50% of individuals over the age of 60 may have asymptomatic RCTs [2].

Despite the high prevalence of RCTs, the accurate, rapid diagnosis and evaluation of the severity of this condition can be challenging [3]. When evaluating RCTs, we generally examine the supraspinatus/infraspinatus tendon complex (SITC) and subscapularis tendon separately. The SITC is the most frequently torn tendon and can cause significant pain and disability for patients. Clinical information such as medical history, physical examination, and clinical scoring can assist in detecting SITC injury with a sensitivity and specificity range of 40 to 80% [4]. Radiographs such as shoulder x-ray are convenient and allow for the visualization of bone changes such as humeral head migration and subacromial spurs that are associated with SITC injuries [5]. And the detection of sclerosis and cortical irregularity at the greater tuberosity through shoulder x-rays could suggest the presence of a SITC injuries. However, it is important to note that x-rays exhibit limited sensitivity when it comes to accurately detecting SITC injuries [6]. Advanced imaging techniques such as ultrasound, magnetic resonance imaging (MRI), or arthrography may be necessary for accurate identification of SITC injuries. However, the accuracy of ultrasound for SITC injuries heavily relies on the clinician’s expertise and experience, with reported sensitivities and specificities varying within the range of 60–100%. On the other hand, MRI or arthrography can be costly and may not be necessary for all patients.

Machine learning and deep learning (DL) neural networks have recently emerged as promising tools for diagnosing and evaluating SITC injuries [7–10]. However, these models have some limitations in clinical application. Firstly, most of these models are developed based on a single modality, either clinical information or MRI, leading to lower diagnostic performance [9, 10]. Secondly, these models lack the ability to comprehensively evaluate RCTs, as clinicians do, by assimilating patients’ clinical information, physical examination, scoring, and radiography findings. Lastly, the potential benefits of utilizing artificial intelligence in actual diagnostic scenarios for medical professionals have not been analyzed. Therefore, accurate and reliable diagnostic tools that can effectively combine clinical information with radiographic findings for early detection and accurate assessment of the severity of RCTs are urgently needed. Such tools align better with the diagnostic thinking habits of clinicians.

The study was devised with these limitations in mind (1) to develop the ensemble CML-DL model, a deep learning model that incorporates clinical information and radiographic findings to accurately assess the severity of SITC injury, and (2) to validate the clinical benefits of using this deep learning model to assist clinical decision-making. We believe that the development and implementation of such tools can improve patient outcomes, reduce the burden of disease, and optimize treatment strategies.

## Methods

### Patients

This retrospective study was conducted in accordance with the Helsinki Declaration and was approved by the ethics committees of all hospitals involved (SYSEC-KY-KS-2021–184). The requirement for informed consent was waived. This study adhered to the Standards for Reporting Diagnostic Accuracy Studies guidelines to ensure accurate and transparent reporting. Additionally, the Transparent Reporting of a Multivariable Prediction Model for Individual Prognosis or Diagnosis Guidelines were followed to provide a comprehensive framework for reporting prediction models in this study.

**Fig. 1:**
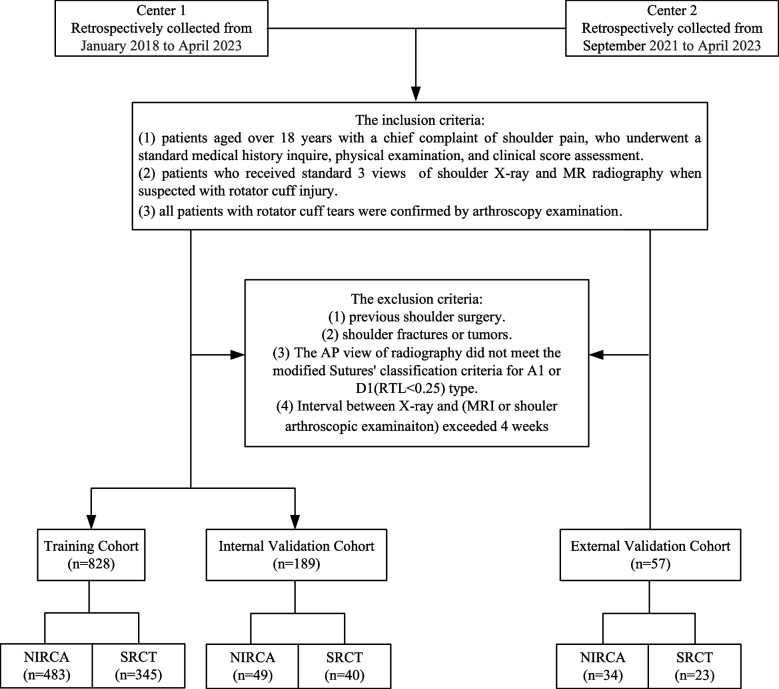
Flowchart of patient enrollment pathway

We selected patients from the Sun Yat-sen Memorial Hospital of Sun Yat-sen University (center 1) as our primary cohort due to its larger sample size. The study included patients enrolled between January 2018 and April 2023. For internal validation, we included patients admitted after January 2021, while others were used for the training cohort. To ensure independent external validation, we also included data from the Shenshan Medical Center, Sun Yat-sen Memorial Hospital of Sun Yat-sen University (center 2), as external validation cohorts (Fig. 1).

This study included patients who met predefined inclusion criteria: (1) patients over 18 year old with a chief complaint of shoulder pain, who underwent a standard medical history inquire, physical examination, and clinical score assessment; (2) patients who received standard anteroposterior view of shoulder x-ray radiograph and MRI when suspected with rotator cuff injury; (3) all patients suspected with SRCT were confirmed by arthroscopy examination; The exclusion criteria were as follows: (1) patients who had undergone previous shoulder surgery; (2) patients with shoulder fractures or tumors; (3) the AP view of radiography did not meet the Suter-Henninger (SH) scapular classification system [11] criteria for A1 or C1 type; and (4) patients with a time interval greater than 4 weeks between clinical assessment and either MRI or shoulder arthroscopy examination.

To analyze the SITC abnormalities, patients were classified into two groups: (1) normal or insignificant rotator cuff abnormalities (NIRCA), encompassing normal rotator cuff, tendinosis, and low-grade partial tears (tears involving ≤ 50% of the tendon thickness), and (2) significant rotator cuff tears (SRCT), which include high-grade partial tears (tears involving > 50% of the tendon thickness) and full-thickness tears, the latter potentially necessitating surgical intervention.

### Clinical information

The clinical information was gathered from a multi-center database as part of a multi-center database of shoulder clinical research program (Table 1). This data included patient demographics (age, gender), trauma history, hand dominance, physical examination outcomes (such as painful arc, pseudoparalysis, Jobe tests, external rotation lag sign, lift-off test, belly press test, bear hug test, internal rotation lag sign, Neer sign, Hawkins-Kennedy test, coracoid impingement test, tenderness, Yergason’s test, and Speed’s test), pain levels assessed by the visual analog scale (VAS) score, and clinical scores including the American Shoulder and Elbow Surgeons (ASES) score, Constant-Murley score, Quick Disabilities of the Arm, Shoulder, and Hand (Quick-DASH) score, Simple Shoulder Test (SST), and University of California, Los Angeles (UCLA) score. During pain level assessments, we recorded the VAS score for the most intense pain, regardless of the patient’s posture or mobility. For missing data, such as history of trauma or dominant hand, we generated a separate “unknown” category.

**Table 1:** Clinical information collected from a multi-center database of shoulder clinical research program

Characteristics	Description
Baseline characteristics	Sex, age, dominant hand, VAS score, trauma history
Range of motion	Forwards flexion, external rotation, internal rotation, and external rotation
Muscle strength	Supraspinatus, infraspinatus, deltoid, biceps, and trapezius
Physical tests	Painful arc, pseudoparalysis, Jobe test, 0°Jobe test, external rotation lag sign, lift-off test, belly press test, bear hug test, internal rotation lag sign, Neer sign, Hawkins-Kennedy test, coracoid impingement test, tenderness, Yergason’s test, and Speed’s test
Clinical score	American Shoulder and Elbow Surgeons (ASES), Constant-Murley score, Quick Disabilities of the Arm, Shoulder, and Hand (Quick-DASH), Simple Shoulder Test (SST), and University of California, Los Angeles (UCLA) score

### Image acquisition and preprocessing

We retrospectively reviewed shoulder pain patients who visited the orthopedic clinic and underwent standard shoulder anteroposterior radiographs. All radiographs were downloaded in anonymized digital imaging and communications in medicine (DICOM) format. Two orthopedic physicians (J.Y.H. and Q.Z., with 5 and 7 years of experience, respectively) reviewed the images. To eliminate irrelevant information from non-lesion areas, we defined a region of interest (ROI) on the radiographs, with a 512 × 512-pixel rectangular area centered on the humeral head, and the ROI rectangle was then cropped.

### Clinical features obtained from CML model

To obtain features from clinical information, we trained four benchmark models—random forest, support vector machine (SVM), lasso, and decision tree—on a cumulative set of 35 clinical variables. The Student *T* test or *U* test method was employed to choose significant features that could differentiate between patients with SRCT or NIRCA. Only features with *p*-values less than 0.05 were kept. The maximum relevance minimal redundancy (mRMR) method was used to assess the relevance and redundancy of each attribute. Maximum relevance sought to identify the attribute with the highest correlation with muscle status. The minimum redundancy criterion was used to ensure that features with the least redundancy were chosen. Using the mRMR technique, the relevance-redundancy index was utilized to order the features. To build the prediction models, a set of significant features with good correlation and low redundancy was chosen.

### Deep visual features obtained from DL model

This study applied different deep learning models to predict SITC injury based on shoulder radiographs. The research employed three benchmark deep learning models, namely Resnet-101, Visual Geometry Group (VGG)-19, and Inception-V3. Prior to the training process, these benchmark models underwent pre-training with the ImageNet repository, which contains over one million images of natural origin and a thousand categories of objects. To customize the deep learning models for our specific task, we executed fine-tuning using transfer learning. This process involved freezing the weights of convolution layers that were initially optimized for identifying structures in images. We then replaced the deep layers with innovative, fully connected task-specific layers that were retrained using the backpropagation algorithm. After fine-tuning the three benchmark DL models, we extracted the deep visual features of shoulder radiographs from the fully connected layers. Finally, we trained four DL models on each subset independently and recorded the predicted results. To maximize the deep visual features and enhance the generalization ability of the model, we generated an ensemble DL model by integrating four DL models into a single ensemble model using advanced feature fusion techniques.

### Ensemble CML-DL scheme

**Fig. 2:**
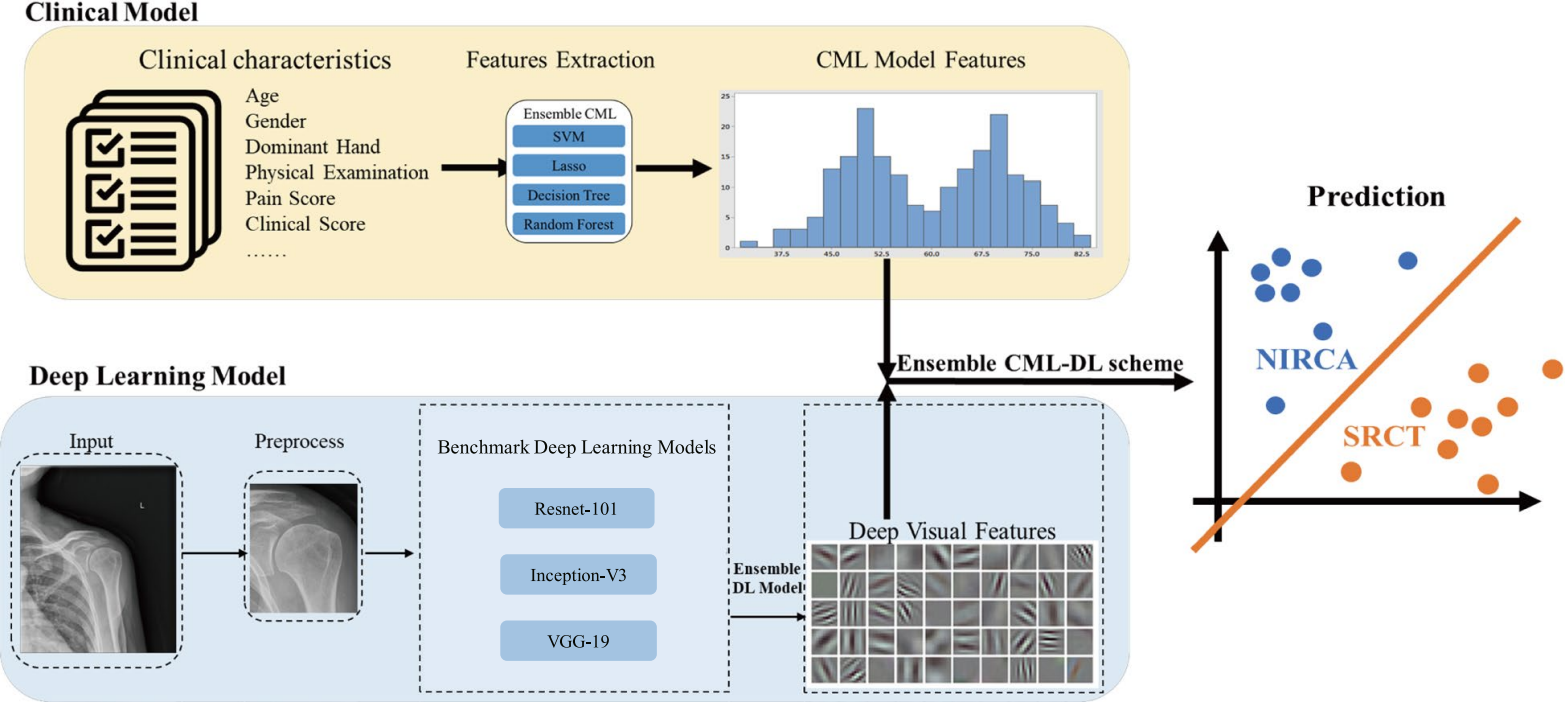
Demonstration of construction of the ensemble CML-DL scheme. Four CML models including SVM, lasso, decision tress, and random forest were trained to obtain features from clinical characteristics. An ensemble DL scheme and three benchmark DL models were used to merge the image features extracted from shoulder radiographs. Finally, an ensemble CML-DL model was used to integrate the features obtained from images and digital data to predict NIRAC and SRCT. SRCT, significant rotator cuff tear; NIRCA, normal or insignificant rotator cuff abnormality; SVM, support vector machine, DL, deep learning; CML, clinical machine learning; VGG, Visual Geometry Group

The CML scheme with the highest AUC was selected to generate the CML-DL scheme by combining the ensemble DL model. The above-mentioned methods utilized integrated features that combined 297 deep visual features, extracted from the ensemble deep learning model, with clinical variables as inputs to predict the outcome for SITC injuries. Our approach effectively leverages the strengths of multiple DL models and demonstrates their potential for accurate and efficient prediction of SITC injuries from shoulder radiographs. Figure 2 illustrates the detailed architecture of the ensemble CML-DL scheme.

### Two-round assessment

To examine the clinical advantages physicians could gain from using the CML-DL model, a two-round assessment was conducted. The study included five physicians affiliated with center 1: three junior physicians (physicians 1–3) with an average clinical experience of 2.3 years (range: 2–3 years) and two experts (physicians 4–5) with an average of 6.5 years of experience in shoulder joint surgeries (range: 5–8 years). An additional cohort of five physicians affiliated with center 2 were also included in the study. The study involved two groups of physicians: physicians 6–8 who were classified as junior physicians with an average of 1.8 years of clinical experience ranging from 1 to 2 years and physicians 9–10 who were classified as experts with an average of 7 years of experience in shoulder joint surgeries ranging from 5 to 9 years. The study examined a total cohort of 146 patients, including 63 individuals who underwent SCRT and were randomly presented from both internal and external validation cohorts. Physicians should make informed decisions based on clinical data and radiographic findings on x-ray films. These may include evidence of a humeral head migration, which suggests a possible SITC injury. Additionally, the presence of subacromial spurs could indicate a potential impingement syndrome, and a critical shoulder angle (CSA) greater than 35° can serve as a risk factor for SITC injury. Throughout the investigation, the medical practitioners were blinded to both the outcomes of the MRI or arthroscopy assessments and each other’s observations.

To assess the diagnostic accuracy of the ensemble CML-DL model, we calculated a total score based on the opinions of five physicians. If a patient was identified as having a significant rotator cuff tear (SRCT) by a physician, one point was awarded. Therefore, the maximum score achievable was 5, while the minimum was 0. A higher total score indicated that a greater proportion of physicians believed the patient had a SRCT.

### Statistical analysis

We evaluated the predictive accuracy of the CML, DL, and CML-DL models for SITC injury by calculating their sensitivity and specificity, plotting ROC curves, and calculating the corresponding AUC values. Statistical analyses were conducted using SPSS (version 22.0) and Python 3.8. Continuous variables were presented as mean ± standard deviation (SD), and categorical variables were presented as numbers and percentages. Between-group comparisons were conducted using Student’s *t*-test or Mann–Whitney *U* test for quantitative variables and the chi-squared test for qualitative variables. The 95% confidence interval (CI) was computed using bootstrapping with 2000 resamples. All statistical analyses were two-tailed, and a *p*-value < 0.05 was considered statistically significant.

## Results

### Clinical information

Based on the inclusion and exclusion criteria, 974 patients from two medical centers were included in the research. Patients from center 1 were split into 828 patients for the training cohort and 89 patients for the internal validation cohort to construct and verify the model. The external validation cohort comprised of 57 patients from center 2. There were no statistically significant differences observed in any characteristics between the NIRCA and SRCT groups in both the internal and external validation cohorts (*p* > 0.05). Table 2 presents the demographic characteristics of these patients.

**Table 2:** Demographic characteristics of patients suspected with rotator cuff tears

Characteristics	Training cohort			Internal validation cohort			External validation cohort		
	**NIRCA (*n* = 483)**	**SRCT (*n* = 345)**	***p***	**NIRCA (*n* = 49)**	**SRCT (*n* = 40)**	***p***	**NIRCA (*n* = 28)**	**SRCT (*n* = 29)**	***p***
Age (years, mean ± SD)	49.94 ± 14.20	57.28 ± 11.32	< 0.001	51.29 ± 13.22	55.50 ± 12.25	0.126	49.94 ± 14.20	57.28 ± 11.32	0.408
Sex			0.013			1			0.9
Female	252 (52.17)	211 (61.16)		27 (55.10)	22 (55.00)		252 (52.17)	211 (61.16)	
Male	231 (47.83)	134 (38.84)		22 (44.90)	18 (45.00)		231 (47.83)	134 (38.84)	
Dominant side			< 0.001			0.361			0.204
Dominant side	237 (49.07)	202 (58.55)		27 (55.10)	22 (55.00)		237 (49.07)	202 (58.55)	
Non dominant side	202 (41.82)	89 (25.80)		11 (22.45)	13 (32.50)		202 (41.82)	89 (25.80)	
Unknown	44 (9.11)	54 (15.65)		11 (22.45)	5 (12.50)		44 (9.11)	54 (15.65)	
Degree of pain			0.002			0.468			0.6
Mild	153 (31.68)	73 (21.16)		13 (26.53)	7 (17.50)		9 (32.14)	5 (17.24)	
Moderate	209 (43.27)	156 (45.22)		23 (46.94)	20 (50.00)		15 (44.12)	10 (43.48)	
Severe	52 (10.77)	41 (11.88)		4 (8.16)	7 (17.50)		5 (14.71)	3 (13.04)	
Unknown	69 (14.29)	75 (21.74)		9 (18.37)	6 (15.00)		8 (23.53)	5 (21.74)	
History of trauma			< 0.001			0.011			0.502
Present	138 (28.57)	148 (42.90)		14 (28.57)	23 (57.50)		12 (35.29)	11 (47.83)	
Absent	345 (71.43)	197 (57.10)		35 (71.43)	17 (42.50)		22 (64.71)	12 (52.17)	
Flex (deg, mean ± SD)	149.25 ± 34.52	150.45 ± 41.46	0.652	146.33 ± 37.06	145.47 ± 49.81	0.927	140.29 ± 38.41	138.65 ± 55.60	0.895
Abd (deg, mean ± SD)	139.82 ± 40.87	143.62 ± 46.21	0.212	136.14 ± 40.61	139.30 ± 53.19	0.752	133.26 ± 39.53	131.39 ± 56.22	0.883
ER (deg, mean ± SD)	49.74 ± 23.96	58.62 ± 19.86	< 0.001	48.80 ± 22.51	58.60 ± 24.45	0.052	45.62 ± 24.15	58.00 ± 26.97	0.075
IR (deg, mean ± SD)	5.43 ± 3.08	4.43 ± 2.62	< 0.001	5.12 ± 2.96	5.95 ± 8.99	0.546	6.15 ± 2.58	7.13 ± 11.61	0.634
ASES (*n*, mean ± SD)	60.27 ± 18.16	53.84 ± 18.82	< 0.001	56.69 ± 16.93	55.75 ± 20.15	0.811	58.53 ± 14.45	57.83 ± 18.71	0.874
Constant-Murley (*n*, mean ± SD)	70.69 ± 14.40	66.24 ± 15.29	< 0.001	69.41 ± 13.92	63.08 ± 19.72	0.08	68.26 ± 12.09	61.22 ± 18.81	0.09
Quick DASH (*n*, mean ± SD)	29.49 ± 15.42	33.34 ± 18.91	0.001	30.61 ± 16.36	34.20 ± 20.70	0.364	32.12 ± 15.66	34.70 ± 21.07	0.598
SST (*n*, mean ± SD)	6.64 ± 2.54	6.17 ± 2.88	0.015	6.14 ± 2.67	7.03 ± 5.75	0.342	5.91 ± 2.37	7.39 ± 6.93	0.254
UCLA (*n*, mean ± SD)	22.22 ± 5.20	21.29 ± 5.82	0.017	22.37 ± 5.68	20.65 ± 6.57	0.19	23.12 ± 4.89	20.30 ± 6.96	0.078

### Model performance of CML, DL, and ensemble CML-DL scheme

Table 3 presents a comparison of diagnostic performance among different models. The differences in performance between different networks in the CML and DL models were small in the internal validation cohort (*p* < 0.05). Among all the DL models, the ensemble DL model demonstrated the highest performance, with a sensitivity, specificity, accuracy, and AUC (95% CI) of 0.800, 0.653, 0.712, and 0.797 (0.734–0.861), respectively.

**Table 3:** Diagnostic performance of the deep learning algorithm

Models	Sensitivity	Specificity	Accuracy	AUC	95% CI
CML models					
Lasso	0.893	0.667	0.763	0.832	0.773–0.890
SVM	0.933	0.627	0.757	0.866	0.815–0.917
Decision tree	0.759	0.753	0.756	0.826	0.795–0.856
Random forest	0.783	0.863	0.829	0.897	0.8750–0.919
DL models					
Resnet-101	0.793	0.596	0.678	0.753	0.717–0.788
VGG-19	0.827	0.637	0.718	0.788	0.721–0.854
Inception-V3	0.793	0.596	0.678	0.753	0.717–0.788
Ensemble DL model	0.8	0.653	0.712	0.797	0.734–0.861
Ensemble CML-DL model	0.88	0.812	0.836	0.902	0.858–0.947

Based on the evaluation of various models, we selected random forest as the basis for the CML-DL scheme, as it achieved the highest AUC. As expected, the results indicate that the CML-DL model outperformed all other CML models, exhibiting a sensitivity, specificity, accuracy, and AUC (95% CI) of 0.880, 0.812, 0.836, and 0.902 (0.858–0.947), respectively.

**Fig. 3:**
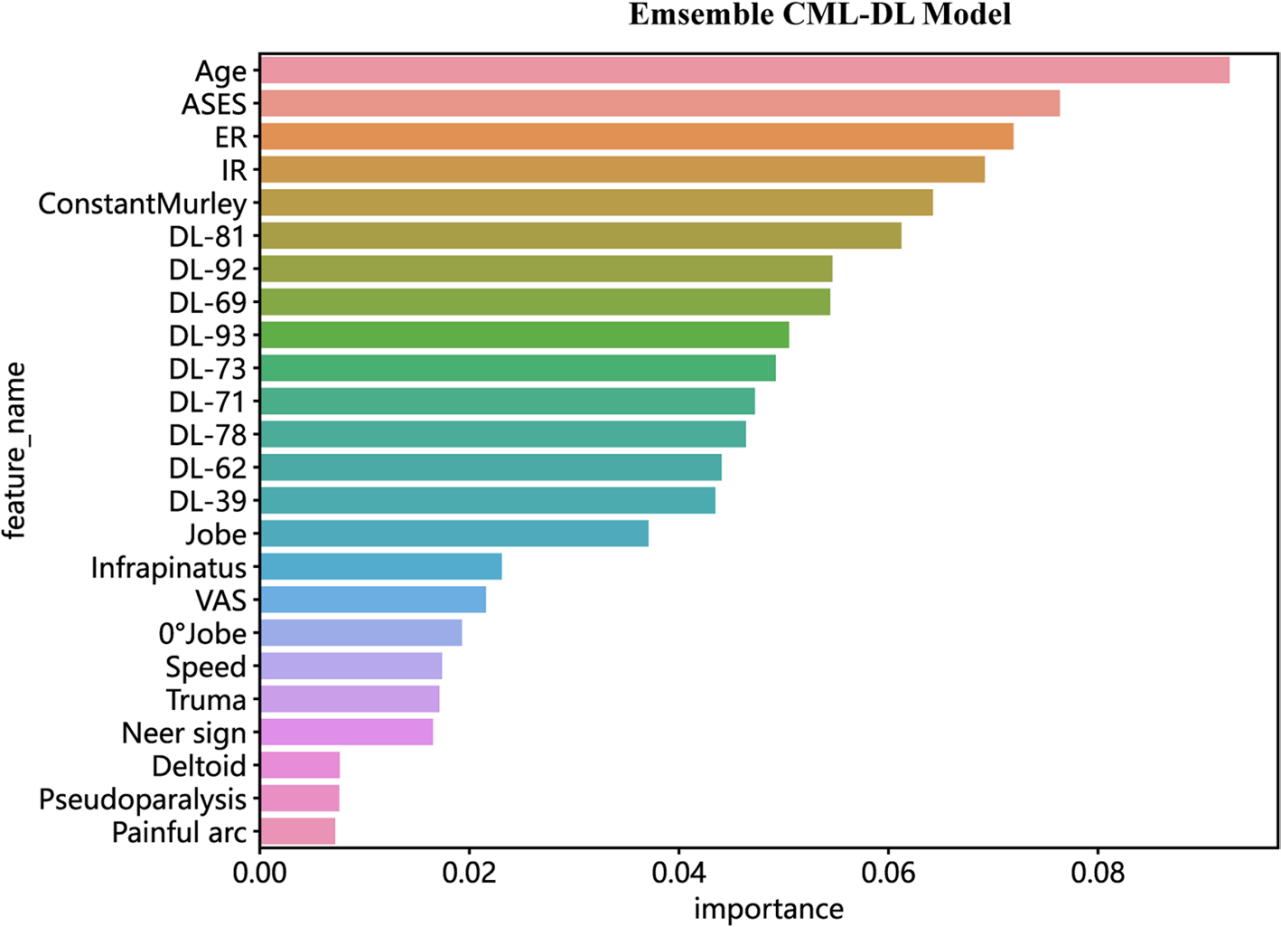
The variance importance plot lists the most significant variables in descending order

The CML-DL model, as an ensemble, has identified 24 features that are significant and have consistently demonstrated prognostic efficacy in predicting SITC injury. Figure 3 displays the top 15 features, including 6 clinical and 9 deep visual features, listed in descending order of significance. These features were found to be highly informative and played important roles in the model’s ability to accurately predict the SITC injury.

### Comparison of the deep learning radiomics model to physicians

**Fig. 4:**
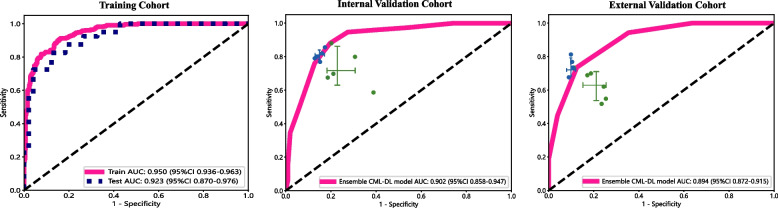
Comparison of the performance of the ensemble CML-DL model with that of physicians. The figure displays the identification of SRCT and NIRCA in the training cohort, internal validation cohort, and external validation cohort using the ensemble CML-DL model and by individual physicians. The performance of our ensemble CML-DL model is compared with each of the five readers and the average reader

In the first-round assessment, we contrasted the diagnostic decisions of five physicians with the ensemble CML-DL model. Figure 4 illustrates the ROC curve of the ensemble CML-DL model, the diagnoses of each physician, and the average diagnostic results of all physicians in the various cohorts. Our result showed the ensemble CML-DL model achieved high AUC values of 0.950 (95% CI 0.936–0.963), 0.902 (95% CI 0.858–0.947), and 0.894 (95% CI 0.872–0.915) in the training, internal validation, and external validation cohorts, respectively. The sensitivity in the internal and external validation cohorts were 88.0% and 73.6%, respectively, while the specificity was 81.2% and 87.5%, respectively.

Our results indicate that the diagnoses provided by the five physicians were either inferior or equivalent to those generated by the CML-DL model ensemble. The ROC curve analysis indicates that the model’s diagnostic accuracy surpassed that of the physicians, as indicated by the conspicuous lack of green points in the upper left region. Furthermore, the average of all five physicians’ diagnoses in all validation cohorts were located below the ROC curve of the ensemble CML-DL model (Fig. 4, green crosses), indicating that our model was superior to the physicians in general.

Moreover, the mean value of the diagnoses provided by the five physicians across all validation cohorts was positioned beneath the ROC curve of the ensemble CML-DL model (Fig. 4). This suggests that our model outperformed the physicians in a general sense.

### Enhanced diagnosis with AI assistance

We analyzed the alterations in diagnoses provided by five physicians before and after AI assistance. The detailed changes in their decision, sensitivity, and specificity are presented in Table 4. The implementation of AI assistance resulted in a significant improvement in the performance of junior physicians (1, 2, and 6–8) across all validation cohorts, without compromising the specificity of the diagnostic process (*p* < 0.05). The results indicate that the ensemble CML-DL model has a favorable impact on the average accuracy of physicians, as evidenced by the blue points and crosses depicted in Fig. 4. Notably, in the second round of evaluation, physicians 5 and 10 (junior) had significantly higher specificity compared to their performance in the first round (*p* < 0.05).

**Table 4:** Summary of the changes in the decision-making of radiologists before and after AI assistance

	**Physician**	**True negative**	**True positive**	**Sensitivity (%)**	**Specificity (%)**
**Internal validation cohort (*n* = 89)**	1	32 → 40	33 → 34	0.660 → 0.790^*^	0.821 → 0.869
	2	36 → 41	18 → 33	0.581 → 0.804^*^	0.621 → 0.854^*^
	3	37 → 42	30 → 32	0.741 → 0.820	0.787 → 0.840
	4	45 → 44	29 → 31	0.878 → 0.961	0.804 → 0.830
	5	42 → 40	28 → 33	0.800 → 0.785	0.778 → 0.851^*^
**External validation cohort (*n* = 57)**	6	22 → 28	15 → 18	0.577 → 0.750^*^	0.742 → 0.848^*^
	7	19 → 25	17 → 17	0.531 → 0.654^*^	0.760 → 0.806
	8	26 → 30	17 → 18	0.680 → 0.818^*^	0.812 → 0.857
	9	28 → 29	18 → 18	0.750 → 0.783	0.848 → 0.853
	10	25 → 25	15 → 19	0.625 → 0.678	0.757 → 0.862^*^

^*^Indicates the *p* < 0.05

**Fig. 5:**
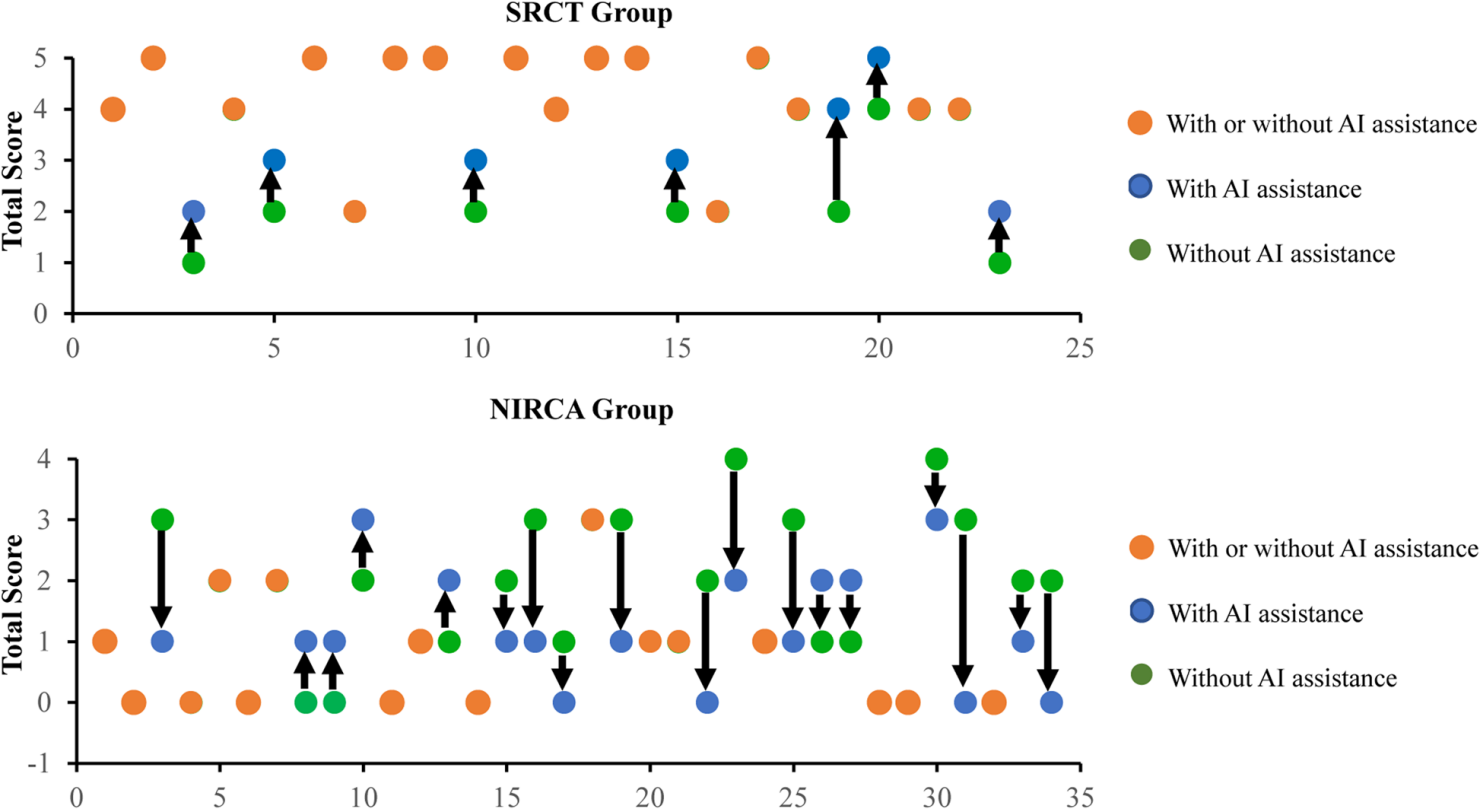
A summary of the total scores from five physicians before and after CML-DL model assistance for each case in the external validation cohort. The green and blue circles represent the total score without and with CML-DL model assistance, respectively. The orange circles indicate that the lesion received the same score before and after AI assistance. The arrows indicate the trend of the total score after AI assistance. The total score is calculated as the sum of the scores of the five physicians individually. If an expert believes that a SRCT is present, it is scored as one point, leading to a maximum score of 5. The higher the score, the more physicians believe that the case is a SRCT. SRCT, significant rotator cuff tear; NIRCA, normal or insignificant rotator cuff abnormality; AI, artificial intelligence

Figure 5 illustrates the total scores of all cases in the external validation cohort, as assessed by five physicians, to demonstrate the clinical value of our ensemble CML-DL model. Although the ensemble CML-DL model’s predictions may have resulted in incorrect decisions by physicians in some cases, the cumulative scores of the five physicians across all cases in the validation cohorts showed a noticeable improvement in diagnostic efficacy after the implementation of ensemble CML-DL assistance.

## Discussion

In this study, our aim was to develop and evaluate the performance of an ensemble CML-DL model for the diagnosis of SITC injuries. Our model demonstrated superior diagnostic performance compared to human physicians in both internal and external validation cohorts. Moreover, we demonstrated that the implementation of the ensemble CML-DL model could improve the diagnostic accuracy of human physicians, highlighting its potential clinical utility in real-world settings.

Our study demonstrated that the ensemble CML-DL model, integrating clinical information and radiography findings, exhibited superior or comparable diagnostic performance to previous studies. This can be attributed to several factors. First, previous studies have shown that medical history, physical examination, and clinical scores are useful for assessing rotator cuff injuries. ML models can improve diagnostic accuracy by automatically learning adaptive features from clinical information [7, 9, 10, 12, 13]. Secondly, radiographic findings are highly correlated with rotator cuff injuries, with specific features such as humeral head migration, supraspinatus calcification, and CSA > 35° being particularly indicative of RCTs. DL models can identify high-level abstract features that human clinicians may not recognize, resulting in higher precision and accuracy [14, 15]. Lastly, ensemble models can achieve better accuracy and generalizability than single deep learning models by combining predictions from multiple models, reducing individual biases and errors, and improving model robustness to overfitting [16].

One of the main strengths of our study was incorporating a real-world assessment with ten physicians from two different centers. This assertion holds significant importance as it is anticipated that CML-DL models will serve as a supplementary component in the coming times. Despite the advantages of DL and radiomics models, ultimate decision-making authority will remain with human physicians. A primary contributing factor to this phenomenon is the nascent stage of interpretability pertaining to deep learning features, coupled with the underexplored nature of the biological mechanism underlying radiomics features. Nevertheless, physicians should not refrain from utilizing deep learning techniques to enhance their diagnostic abilities. The prediction score of the ensemble CML-DL model functioned as a reliable signal for the physicians involved in this study. The model’s outlier score, which indicated high confidence in classifying lesions as either NIRCA or SRCT, played a crucial role in notifying medical practitioners regarding patients with diverse diagnoses determined by both human interpretation and quantitative computational analysis. When this assistance approach was used during the second round of image interpretation, physicians had a remarkable enhancement in their capacity to precisely identify and evaluate SRCT while still maintaining specificity. The ensemble CML-DL model has the potential to benefit clinical practice by supporting junior physicians. Although all physicians received valuable assistance from the model, junior physicians experienced a more significant benefit. Consequently, this methodology can enhance the learning rate of physicians with limited experience.

Despite the promising results of our study, there were several limitations that should be acknowledged. Firstly, the retrospective nature of our study resulted in missing patient information and the exclusion of many patients who did not meet our inclusion and exclusion criteria. Future prospective clinical studies could be conducted to further validate the performance of our ensemble CML-DL scheme. Secondly, although our study was conducted across multiple centers, the dataset used in this study was small. The use of strict inclusion and exclusion criteria, which required specific shoulder radiographs to be eligible for inclusion, resulted in smaller sample sizes at each center and restricted the number of centers that met our eligibility criteria. Lastly, only standard AP view of shoulder radiographs were included in this study. Future studies could consider including additional radiographic views, such as the Y-view and Stryker notch view, which may enhance the model’s predictive accuracy. Nevertheless, the strong performance of our model demonstrated its effectiveness in assisting clinicians to improve the diagnosis of rotator cuff injuries. Future studies with larger, more diverse datasets, multiple imaging views, and prospective designs are needed to further validate and extend our findings.

## Conclusions

Our study successfully established an ensemble CML-DL model by combining clinical and deep visual features. The ensemble CML-DL model provides a valuable solution to help physicians improve the diagnostic performance of SITC injury, particularly for junior physicians with limited expertise.

## Data Availability

All data used or analyzed in this study are available from the corresponding author upon reasonable request.

## Abbreviations

CML: Clinical machine learning
DL: Deep learning
MRI: Magnetic resonance imaging
NIRCA: Normal or insignificant rotator cuff abnormality
RCTs: Rotator cuff tears
SITC: Supraspinatus/infraspinatus tendon complex
SRCT: Significant rotator cuff tear
VAS: Visual analog scale
